# XEN Gel Stent Implantation in Eyes With Previous Glaucoma Filtering Surgeries: A Case Series

**DOI:** 10.7759/cureus.32741

**Published:** 2022-12-20

**Authors:** Rita Vieira, João Leite, Ana Figueiredo, Rita Reis, Isabel Sampaio, Maria João Menéres

**Affiliations:** 1 Ophthalmology, Centro Hospitalar Universitário do Porto, Porto, PRT

**Keywords:** migs, xen gel stent, second intention surgery, minimal invasive glaucoma surgery, xen gel implant

## Abstract

Background

In this study, we aimed to analyze the efficacy and safety of *ab interno *XEN^®^ gel stent (Allergan Inc., Dublin, Ireland) implantation in a series of eyes with open-angle glaucoma and a history of previous filtering glaucoma surgeries.

Methodology

This retrospective, single-center study included all eyes that underwent XEN gel implantation with a minimum follow-up of 18 months that had previously undergone a filtering glaucoma surgery. The main outcomes were intraocular pressure (IOP) variation (baseline, first day, first week, and first, third, sixth, 12th, and 18th months), the number of hypotensive eyedrops at 18 months, intraoperative and postsurgical complications, and the need for subsequent glaucoma surgery.

Results

A total of 10 eyes from nine patients were enrolled in the study. IOP significantly decreased from 24.0 ± 2.4 mmHg to 7.8 ± 1.6 mmHg on the first day, 9.4 ± 2.2 mmHg in the first week, and 12.3 ± 4.8 mmHg, 14.7 ± 4.3 mmHg, 13.3 ± 3.8 mmHg, 11.7 ± 1.9 mmHg, and 12.3 ± 1.9 at the first, third, sixth, 12th, and 18th month, respectively (p < 0.001), with a 49.5% reduction in IOP at the end of the follow-up. The number of hypotensive eyedrops decreased significantly from 3.5 ± 0.5 (3-4) to 0.7 ± 0.7 (0-2) (p = 0.026). No intraoperative or postsurgical complications were recorded. In total, seven (70%) eyes achieved controlled IOP of <18 mmHg without any anti-glaucomatous medications, and one (10%) eye with the use of topical prostaglandin. One (10%) eye was submitted to a surgical revision, and another needed an additional glaucoma surgery during follow-up, with appropriate IOP control at the last follow-up.

Conclusions

This case series aims to highlight that the XEN implant can be an option in eyes with IOP levels in the low 20s and previously failed filtering surgeries, with satisfactory efficacy and high safety.

## Introduction

Glaucoma is a chronic, progressive optic neuropathy that is considered to be one of the leading causes of blindness worldwide. Untreated cases of uncontrolled intraocular pressure (IOP) usually result in severe vision loss [1,2]. The main goal of glaucoma treatment is lowering the IOP, which can be achieved by hypotensive topical drugs and surgery. Although hypotensive eyedrops are highly effective, glaucoma surgery is indicated when IOP remains uncontrolled with maximum topical medication, when progression is documented in structural or functional examinations, in cases of pharmacological intolerance, or as a patient’s option, particularly if there is poor pharmacological compliance [1,3].

Trabeculectomy remains the gold-standard surgical option for the management of glaucoma, with a significant and sustained decrease in IOP [4]. Other filtering surgeries, such as deep sclerectomy and ExPRESS implant, are widely used as alternatives to trabeculectomy. All filtering surgeries create a bypass of aqueous humor flow from the anterior chamber to the subconjunctival space, creating a scleral flap that leads posteriorly to the development of a filtration bleb. However, these filtering procedures, particularly trabeculectomy, are associated with considerable postsurgical complications, including cataract development, hypotony, hyphema, choroidal detachment, choroidal effusion or hemorrhage, conjunctival scarring, and endophthalmitis [4,5].

Minimally invasive glaucoma surgery (MIGS) has gained popularity due to its effective reduction of IOP, lower complication rates, minimal tissue disruption, and shorter surgical duration when compared to filtering surgeries [6]. XEN® gel stent (Allergan Inc., Dublin, Ireland) consists of a collagen tube with a 6 mm length and 45 μm inner lumen. It is one of the subconjunctival MIGS devices which creates an alternative outflow pathway of the aqueous humor to the subconjunctival space. Despite being a filtration bleb-dependent procedure such as trabeculectomy, its advantages include the *ab interno* implantation, sparing conjunctiva dissection, scleral flap creation, and the need for an iridectomy. It is a sutureless procedure and reduces postsurgical complications. However, IOP reduction is not as effective as in trabeculectomy [7].

This technique is recommended essentially as a primary intention surgery in mild-to-moderate open-angle glaucoma. Recently, some studies have been published concerning XEN implants in eyes previously submitted to filtering surgeries [8-14]. Therefore, this study aimed to analyze the efficacy and safety of XEN gel implantation in a series of patients who had undergone a filtering glaucoma surgery in the past.

## Materials and methods

This study was conducted in accordance with the Declaration of Helsinki (1964) and its latest amendment (Brazil, 2013). All included patients provided verbal and written consent to participate in the study. The study protocol complied with the requirements of the institute’s committee on human research (Departamento de Ensino, Formação e Investigação).

This was a retrospective, single-center, observational study. All patients with primary or secondary open-angle glaucoma submitted to XEN implantation and a history of a previous failed filtering glaucoma surgery were included. Inclusion criteria included a minimum follow-up period of 18 months, age of at least 18 years, uncontrolled IOP with maximally tolerated hypotensive eyedrops, corrected distance visual acuity of at least hand motion, and absence of conjunctival scarring observed preoperatively on slit-lamp examination. Only solo procedures were included in this study (without concomitant cataract surgery).

Surgical technique

The surgeries were performed by two experienced glaucoma consultants (MJM and IS). The same technique was used in all patients. Surgery was performed under topical anesthesia with oxybuprocaine and intracameral lidocaine. After sterile measures and the placing of an eyelid speculum, the superior nasal conjunctiva was marked 3 mm from the limbus in the planned exit point of the stent. Then, a subconjunctival injection of 0.1 mL of 0.02% diluted mitomycin-C was administered with a 30-gauge needle along with a posterior massage of the area using cellulose sponges, keeping it away from the limbus. The main incision was made temporally, 180 degrees from the exit point, and a side port was placed between the main incision and exit points. A viscoelastic (1% sodium hyaluronate, Provisc-Alcon) was injected into the anterior chamber. The preloaded injector passed through the temporal incision in the direction of the superior nasal quadrant, passing through the trabecular meshwork. When the injector needle was observed subconjunctivally, the implant was injected. An intraoperative gonioscopy was performed to confirm the placement of the implant in the iridocorneal angle [1-3].

Data collection

Baseline sample characterization was achieved by collecting demographic data (age and gender), type of glaucoma, type of previous glaucoma filtering surgeries, central corneal thickness (CCT), endothelium cell count (ECC), retinal nerve fiber layer thickness (RNFLT) of the optic nerve head measured by spectral-domain optical coherence tomography (SD-OCT), visual field index (VFI) using Humphrey static perimetry, best-corrected visual acuity (BCVA) using the Snellen scale, IOP, and the number of anti-glaucoma medications.

The main outcomes evaluated in this study included IOP variation from baseline to the 18th month after surgery, the number of hypotensive eyedrops used to control IOP 18 months after the procedure compared to baseline, the need for additional glaucoma surgery, and surgery-related complications. IOP was measured by Goldmann applanation tonometry on the first day, the first week, and at one, three, six, 12, and 18 months after implantation. Secondary outcomes included variation in BCVA, RNFLT, and ECC 18 months after the surgery. 

Surgical success was defined as IOP ≤18 mmHg or a 20% decrease in IOP compared to baseline: complete success if no hypotensive medications were needed, and relative success if IOP control needed up to two anti-glaucoma medications. Patients with medically uncontrolled IOP were advised an additional IOP-lowering surgery.

Statistical analysis

A descriptive statistical analysis was performed using SPSS version 27.0 (IBM Corp., Armonk, NY, USA). Continuous variables were expressed as mean ± standard deviation (SD) and interval of minimum and maximum values. Non-parametric tests, specifically the Wilcoxon test, were performed to compare dependent continuous variables over time. A p-value of 0.05 was considered significant.

## Results

A total of 10 eyes from nine patients were enrolled, with a mean follow-up of 18.8 ± 0.9 months (18-20). The mean age was 68.3 ± 24.8 years (21-88), and four patients were female (44.4%). Concerning the type of glaucoma, seven (70%) eyes had primary open-angle glaucoma, two (20%) eyes had pseudoexfoliative secondary open-angle glaucoma, and one (10%) eye had juvenile glaucoma. Regarding previous filtering surgeries, eight (80%) eyes had undergone a trabeculectomy, including the patient with juvenile glaucoma; one (10%) eye had a previous deep sclerectomy, and one (10%) eye had an ExPRESS implant. The mean RNFLT was 63 ± 15 mm (37-79), the mean CCT was 512 ± 39 mm (463-577), the mean ECC was 1,781 ± 385 cells/mm^2^ (1,279-2,170), the mean VFI was 66 ± 28% (25-93), and the mean BCVA was 20/32 (20/200-20/20). Eight eyes (80%) were pseudophakic and two were phakic (20%). Baseline data are represented in Table 1.

**Table 1:** Baseline data of the patients. POAG = primary open-angle glaucoma; BCVA = best-corrected visual acuity; RNFL = retinal nerve fiber layer; CCT = central corneal thickness; ECC = endothelium cell count; VFI = visual field index; IOP = intraocular pressure

Number of eyes (n)	10 eyes; 9 subjects
Gender	4 females (44.4%)
	5 males (55.6%)
Age	68.3 ± 24.8 years (21–88)
Type of glaucoma	POAG (n = 7, 70%)
	Pseudoexfoliative (n = 2, 20%)
	Juvenile (n = 1, 10%)
Type of previous filtering surgeries	Trabeculectomy (n = 8, 80%)
	ExPRESS implant (n = 1, 10%)
	Deep sclerectomy (n = 1, 10%)
BCVA (Snellen)	20/32 (20/200–20/20)
RNFL thickness (1 mm central)	63 ± 15 mm (37–79)
CCT	512 ± 39 mm (463–577)
ECC	1,781 ± 385 cells/mm^2^ (1,279–2,170)
VFI	66 ± 28% (25–93)
IOP	24.0 ± 2.4 (20–26) mmHg
Number of hypotensive drugs	3.5 ± 0.5 (3–4)

**Figure 1:**
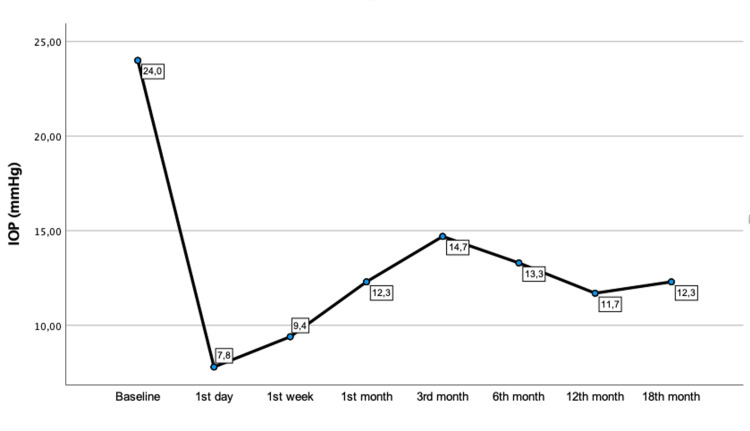
IOP variation during the follow-up (in mmHg). IOP = intraocular pressure

IOP decreased significantly from 24.0 ± 2.4 (20-26) mmHg to 7.8 ± 1.6 (6-10) mmHg on the first day, 9.4 ± 2.2 (6-12) mmHg in the first week, and 12.3 ± 4.8 (8-22) mmHg, 14.7 ± 4.4 (11-24) mmHg, 13.3 ± 3.8 (10-21) mmHg, 11.7 ± 1.9 (10-15) mmHg, and 12.3 ± 1.9 (10-16) mmHg at the first, third, sixth, 12th, and 18th month, respectively (p < 0.001). There was a 49.5% reduction in IOP values by the end of the follow-up compared to the baseline. Figure 1 represents the variation in IOP during the follow-up.

**Figure 2:**
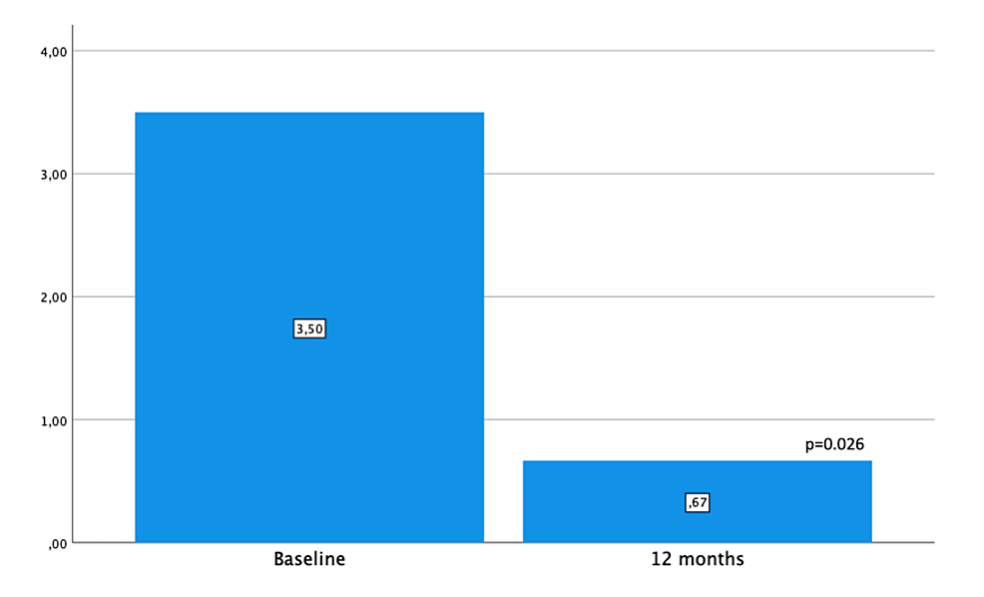
The mean number of hypotensive drugs.

The number of hypotensive eyedrops decreased significantly from 3.5 ± 0.5 (3-4) to 0.7 ± 0.7 (0-2) (p = 0.026), as shown in Figure 2.

No intraoperative complications were recorded. Moreover, there were no postsurgical complications, including hypotonia or hyphema.

In total, seven (70%) eyes achieved absolute success, with IOP under 18 mmHg without medication 12 months after XEN implantation. One (10%) eye achieved relative success, with IOP ≤18 mmHg with one anti-glaucoma medication (prostaglandin analogs, PG). One (10%) eye underwent a surgical revision three months after the first surgery, and one (10%) eye underwent an additional glaucoma surgery during the follow-up. This last patient showed sustained ocular hypertension (OHT) since the first postoperative month, even with topical medication, and underwent an ExPRESS implant three months after XEN surgery. IOP was controlled with ExPRESS and two hypotensive eyedrops (PG and carbonic anhydrase inhibitor).

The patient who underwent a surgical revision showed OHT three months after the surgery. On biomicroscopy, a flat bleb was observed, and the patient started massages and a topical PG. Six months after XEN implantation and with objectively sustained borderline IOP even with a topical PG, the patient was advised a surgical review. During the surgery, it was noticed that the implant was not functioning and a new one was implanted. After this second procedure, the patient exhibited controlled IOP without topical medication until the last follow-up 15 months after the revision.

BCVA remained stable after 18 months (mean final BCVA = 20/32 (20/63-20/20), p = 1.00). No changes were observed in RNFLT (63 ± 17 mm, p = 0.893). There was no endothelium cell loss (ECC = 1,737 ± 510 cells/mm^2^) 18 months after the surgery (p = 0.586).

## Discussion

Although it has been widely studied for mild-to-moderate glaucoma, refractory glaucoma with previous surgeries can be an indication for XEN implantation; however, there is a lack of literature regarding this.

It is known that eyes that previously underwent filtration surgeries may have severe conjunctival scarring despite the use of intraoperative anti-metabolites [15-17].

In eyes with a previously failed trabeculectomy, repeat trabeculectomy is frequently performed with variable success rates, satisfactory qualified success, and relatively few complications [15-17]. A higher need for medications after repeated trabeculectomy has been reported when compared to naïve eyes that underwent trabeculectomy [17]. A recent study by Jagannathan et al. [15] retrospectively reviewed 113 eyes that underwent trabeculectomy alone or associated with phacoemulsification after previously failed trabeculectomy. Their primary outcome was IOP evaluation 12 months after surgery defined by the following three types of success criteria: (A) IOP ≤21 mmHg with ≥20% reduction from baseline, (B) IOP ≤17 mmHg with ≥20% reduction from baseline, and (C) IOP ≤14 mmHg. Although they showed a complete and qualified success of 58.4% and 85.8% with criteria A, respectively, only 36.3% of the eyes maintained an IOP ≤14 mmHg without medication. In addition, the IOP target of 21 mmHg appears to be a borderline result to be considered as a success rate, particularly after a repeated trabeculectomy. Despite no significant postsurgical complications, 66.4% of the patients required hypotensive medications, and 18 (15.9%) eyes had surgical failure.

It is known that the most common causes of surgical failure are subconjunctival and episcleral fibrosis [11,15]. XEN device implantation can be an option in such cases because this procedure does not require conjunctival or scleral dissection. In addition, XEN is usually placed in the superior nasal quadrant, differently from the valves (which are usually placed in the superior temporal quadrants) and from other filtration surgeries, which are generally performed at the 12 o’clock position.

In their recent multicentered case series of seven eyes, Franco et al. [12] reported good results nine months after the XEN implant. In this study, IOP reduction was 41.5%. Only one patient needed additional intervention (bleb needling), and only two patients needed one hypotensive medication to control IOP. Karimi et al. [9] retrospectively reviewed 259 eyes that underwent XEN implantation, where 18 eyes had previously undergone a glaucoma surgery (11 a trabeculectomy and seven an Ahmed valve), and found a similar efficacy in IOP reduction. There was no difference in the number of anti-glaucoma medications after 12 months and similar rates of complications and bleb needling. In another study, Karimi et al. [13] published the first-to-date multicenter case series of 17 eyes that had undergone XEN implantation after a failed trabeculectomy. One year after XEN implantation, there was a significant reduction in IOP and the number of hypotensive eyedrops, with only two (11.8%) eyes requiring a secondary filtration surgery. Nevertheless, a bleb revision was performed in nine (52.9%) eyes, including bleb needling or subconjunctival injection of an anti-metabolite, mainly performed on slit-lamp in the first postoperative month. Despite being a relatively easy procedure showing good results in this group, it is important to remember that bleb needling is not free of hypotony and endophthalmitis risks, especially when performed out of the operating room.

In the only case that underwent a surgical revision, a bleb needling was considered before the decision was made to implant a second XEN. However, this option was discharged in the operating room when a non-functioning obstructed device was noticed. Nevertheless, it is possible to manage obstructed devices in a more conservative manner. In a recent report, Tatti et al. [18] described how they successfully managed an obstructed XEN implant using an *ab interno* approach, with a 25-G vitreous scissor. Despite being a possible and easy approach to address this problem, in their case, a clot was visualized at the proximal edge of the device, a fact that was not similar to our case, whose obstruction was more distal and more difficult to manage.

Despite the good results achieved in some case series, only one study to date performed a direct comparison of XEN implants in naïve eyes and in eyes with previous glaucoma surgeries [11]. Lewczuk et al. compared two groups of 43 eyes, showing no differences in IOP reduction 18 months after surgery (29% in naïve vs. 27% in previously operated eyes) and in the number of hypotensive drugs. Similar to the study by Karimi et al., a high rate of needling was described (70% in naïve vs. 74% in previously operated eyes).

The retrospective design, a relatively short follow-up period, and the low number of eyes are some of the limitations of this study. Prospective studies with larger samples and longer follow-ups are necessary to confirm the place of this procedure in refractory glaucoma with previous surgeries. Comparative studies of XEN versus trabeculectomy and other filtering surgeries after a failed filtering surgery should be performed.

## Conclusions

With this case series, we aim to highlight that XEN implantation can be a reliable option, with satisfactory efficacy and high safety, in eyes with IOP in the low 20s with previous failed filtering surgeries. Its minimally invasive nature and minimal tissue disruption may confer higher rates of success than other filtering procedures such as trabeculectomy in previously operated eyes.
